# Association between brain-derived neurotrophic factor genetic polymorphism Val66Met and susceptibility to bipolar disorder: a meta-analysis

**DOI:** 10.1186/s12888-014-0366-9

**Published:** 2014-12-24

**Authors:** Zuowei Wang, Zezhi Li, Keming Gao, Yiru Fang

**Affiliations:** Division of Mood Disorders, Hongkou District Mental Health Center of Shanghai, Shanghai, 200083 P. R. China; Department of Neurology, Renji Hospital, Shanghai Jiao Tong University School of Medicine, Shanghai, 200127 P. R. China; Department of Psychiatry, Mood and Anxiety Clinic in the Mood Disorders Program, University Hospitals Case Medical Center/Case Western Reserve University School of Medicine, Cleveland, Ohio 44106 USA; Division of Mood Disorders, Shanghai Mental Health Center, Shanghai Jiao Tong University School of Medicine, Shanghai, 200030 P. R. China

**Keywords:** Bipolar disorders, Brain-derived neurotrophic factor, Val66Met, Polymorphism, Case–control, Meta-analysis

## Abstract

**Background:**

In view of previous conflicting findings, this meta-analysis was performed to comprehensively determine the overall strength of associations between brain-derived neurotrophic factor (BDNF) genetic polymorphism Val66Met and susceptibility to bipolar disorders (BPD).

**Methods:**

Literatures published and cited in Pubmed and Wanfang Data was searched with terms of ‘Val66Met’, ‘G196A’, ‘rs6265’, ‘BDNF’, ‘association’, and ‘bipolar disorder’ up to March 2014. All original case–control association studies were meta-analyzed with a pooled OR to estimate the risk and 95% confidence interval (CI) to reflect the magnitude of variance.

**Results:**

Twenty-one case–control association studies met our criteria for the meta-analysis. Overall, there was no significant difference in allelic distribution of Val66Met polymorphism between patients and controls with a pooled OR = 1.03 (95% CI 0.98, 1.08) although there was a trend towards association between Val66Met polymorphism and BPD in Caucasians with an OR of 1.08 (95% CI 1.00, 1.16). However, subgroup analyses showed that there was a significant association of Val allele with decreased disease susceptibility for bipolar disorder type II with a pooled OR of 0.88 (95% CI 0.78, 0.99).

**Conclusions:**

There is no compelling evidence to supportVal66Met polymorphism in BDNF gene playing an important role in the susceptibility to BPD across different ethnicities.

**Electronic supplementary material:**

The online version of this article (doi:10.1186/s12888-014-0366-9) contains supplementary material, which is available to authorized users.

## Background

Bipolar disorders (BPD) are chronic, recurrent, debilitating disorders with high lifetime prevalence and significant disease burden across different populations [1-3]. However, recent advances in pharmacological treatment for BPD remained quite modest. The treatment of bipolar depression is still a major challenge [4,5]. Moreover, BPD is frequently unrecognized and misdiagnosed, particularly in patients presenting with their first-episode of depression. These patients are often treated with inappropriate and costly regimens [6-9]. Thus, there is an urgent need to understand the pathophysiology of BPD in order to develop earlier diagnoses and more effective treatments [10]. Family, twins and epidemiological studies unequivocally demonstrate that BPD is a highly heritable disease with a heritability of more than 85%, and involves the interaction of multiple genes or more complex genetic mechanisms [11-13]. To date, association studies support a possible role for several candidate genes in BPD, including brain-derived neurotrophic factor (BDNF), but consistent direction of effects and alleles have not been established [14]. These inconsistent findings from previous genetic association studies may be related to variation in ascertainment, phenotype definition and control selection, limited power and possibly confounded by ethnic heterogeneity and population substructure [10,15].

The hypothesis of neuronal plasticity involved in mood disorders has been supported by the use of antidepressants and mood stabilizers, e.g. lithium and valproate, inducing the expression of neurotrophins (e.g. BDNF) and synaptic changes [16,17]. Moreover, BDNF gene has been implicated in the etiology of BPD by linkage studies [18]. Position 196 in exon 5 of the BDNF gene contains a G to A transition (dbSNP: rs6265) that results in an amino acid substitution (valine to methionine) at codon 66 in the precursor BDNF peptide sequence [19]. This change results in BDNF functional polymorphisms in this region as Val66Val, Val66Met, and Met66Met. Two previous family-based association studies found that this functional polymorphism Val66Met was significantly associated with the susceptibility to BPD [20,21]. Following these two reports, a large number of association studies between BDNF gene polymorphisms and BPD have been published. Most of them specifically focused on the Val66Met polymorphism, but yielded conflicting results [10,15,22]. In view of the conflicting results, a meta-analysis on all original case–control association studies was performed to comprehensively determine the overall strength of associations between BPD and Val66Met polymorphism.

## Methods

### Literature search

Studies included in the analysis were searched from two databases: Pubmed (http://www.ncbi.nlm.nih.gov/pubmed/) and Wanfang Data (http://www.wanfangdata.com/), with the keywords ‘Val66Met’, ‘G196A’, ‘rs6265’, ‘BDNF’, ‘association’, and ‘bipolar disorder’ in varying combinations. The retrieved abstracts were used to identify studies that examined the allelic association between the Val66Met polymorphism of BDNF and bipolar disorder. Bibliographies or citations from retrieved articles were also cross-referenced as well. The searched period was from the first data available in each database up to March 2014. Two independent authors extracted the following data from each eligible study: last name of the first author, year of publication, ethnicity, sample sizes and allele frequencies of cases and controls, etc. Discrepancies were resolved by mutual consent.

All eligible studies were determined against the following inclusion criteria: (i) published in a peer-reviewed journal; (ii) presented original data; (iii) provided either allele frequency of Val(G)/Met(A), or genotypes (Val/Val, Val/Met, Met/ Met) in both BPD patients and healthy controls; (iv) enrolled more than 100 subjects in both patients group and controls group; and (v) designed as a case–control study. Both family-based studies and genome-wide association studies were excluded in this research. Duplications were deleted, as well as studies that reported all or part of their data previously. The authors of studies were contacted for additional information (e.g. allele or genotype frequencies or characteristics of the samples) if there was uncertainty about whether their data met our inclusion–exclusion criteria, or if we needed additional data which were not contained in the original report.

### Statistical analyses

Data were classified by diagnostic category (case or control) and allele (Val or Met), and Val was assigned as the risk allele. Meta-analysis was performed similar to that described previously [22]. The pooled OR was calculated according to the methods of DerSimonian [23], and its 95% confidence interval (CI) was constructed using Woolf’s method [24]. The Cochran chi-square-based Q statistical test was performed to assess the heterogeneity of ORs, and the significance of the pooled OR was determined by the z-test. If the result of the heterogeneity test was p ≥ 0.05, ORs were pooled according to the fixed-effects model (Mantel-Haenszel methods); otherwise, the random-effects model was used. All statistical analyses were conducted using Review Manager Version 5.2 (RevMan 5.2) [25]. A sensitivity analysis of one-study removed strategy was used to evaluate whether or not the results are being driven by any one specific study, and a funnel plot was used to detect whether or not there is evidence of publication bias. Statistical tests were two-tailed, and the significance level was set at P < 0.05, unless stated otherwise.

## Results

**Figure 1:**
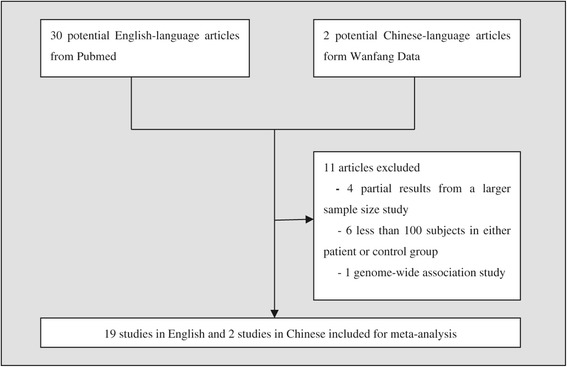
**Identification of studies for meta-analysis.**

The process of identifying studied included in this meta-analysis is shown in Figure 1. Twenty-one case–control association studies met our criteria for the meta-analysis (Table 1). Data from four studies [26-29] were excluded due to the partial overlap with a larger sample size case–control study [30], and data from six studies were excluded due to less than 100 subjects in either patient group or control group [31-36]. Additionally, the case–control sample from a genome-wide association study was also excluded from current meta-analysis [37].

**Table 1:** **Descriptive characteristics of included association studies between BDNF gene Val66Met polymorphism and bipolar disorders**

**Study**	**Year**	**Ancestry**	**Diagnostic criteria**	**Patient’s phenotype**	**Cases**	**Controls**	**Case Val**	**Controls Val**
Hong et al. [38]	2003	Han Chinese	DSM-IV	BPD	108	392	118	406
Nakata et al. [39]	2003	Japanese	DSM-IV	BPI + BPII	130	190	152	220
Kunugi et al. [40]	2004	Japanese	DSM-IV	BPI + BPII	519	588	615	702
Oswald et al. [41]	2004	Caucasian	DSM-IV	BPAD	108	158	166	247
Skibinska et al. [42]	2004	Caucasian	DSM-IV	BPAD	352	375	588	613
Lohoff et al. [43]	2005	Caucasian	DSM-IV	BPI	621	998	1020	1576
Neves-Pereira et al. [44]	2005	Caucasian	DSM-IV	BPAD	263	350	417	547
Schumacher et al. [45]	2005	Caucasian	DSM-IV	BPAD	281	1097	456	1778
Green et al. [46]	2006	Caucasian	DSM-IV	BPI + BPII + Rapid-cycling BPD	1093	2100	1808	3404
Liu et al. [47]	2007	Han Chinese	ICD-10	BPAD	100	100	114	99
Tramontina et al. [48]	2007	Caucasian	DSM-IV	BPI	114	137	183	230
Kim et al. [49]	2008	Korean	DSM-IV	BPD	169	251	186	268
Tang et al. [50]	2008	Han Chinese	DSM-IV	BPD	197	208	238	235
Vincze et al. [51]	2008	Caucasian	DSM-IV	BPD	336	313	532	473
Ye et al. [52]	2009	Han Chinese	DSM-IV	BPD	222	357	217	367
Hosang et al. [53]	2010	Caucasian	ICD-10	BPD	488	598	780	983
Xu et al. [54]	2010	Han Chinese	DSM-IV	BPI + BPII	498	501	525	546
Min et al. [55]	2012	Korean	DSM-IV	BPD	184	214	222	245
Wang et al. [56]	2012	Han Chinese	DSM-IV	BPI + BPII	337	386	341	436
Chang et al. [30]	2013	Han Chinese	DSM-IV	BPI + BPII	967	349	962	361
Pae et al. [57]	2012	Korean	DSM-IV	BPD	132	170	150	197

**Figure 2:**
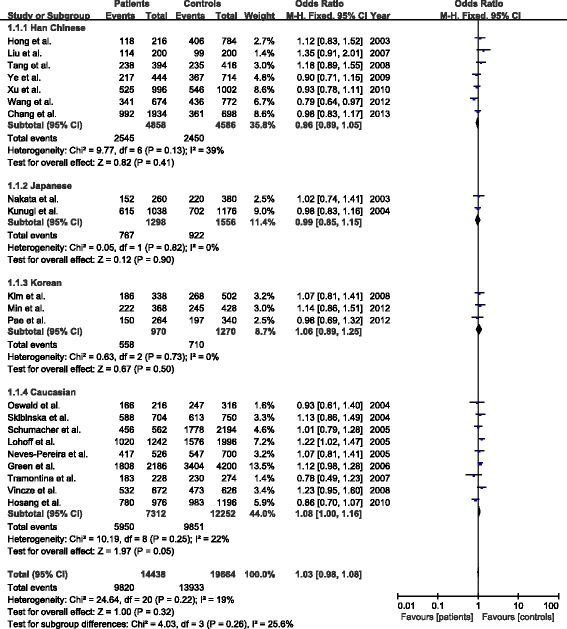
**Fo** **rest plot of association between BDNF gene Val66Met polymorphism and bipolar disorders.**

**Figure 3:**
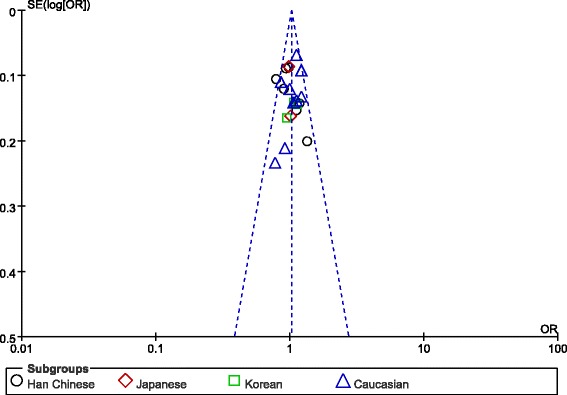
**Funnel plot of publication bias among the 21 included studies.**

Overall, the data from 7219 BPD cases and 9832 healthy controls were analyzed. The mean genotype distribution in Caucasian and Oriental population was presented in Table 2. There was no significant difference in allelic distribution of Val66Met polymorphism between patients and controls. The pooled OR was 1.03 (95% CI: 0.98-1.08, Z = 1.00, P = 0.32) (Figure 2). Similarly, there was also no significant difference in allelic distribution of Val66Met polymorphism between patients and controls in Oriental population, with a pooled OR of 0.96 (95% CI: 0.89-1.05, Z = 0.82, P = 0.41) for Han Chinese population, 0.99 (95% CI: 0.85-1.15, Z = 0.12, P = 0.90) for Japanese population, and 1.06 (95% CI: 0.89-1.25, Z = 0.67, P = 0.50) for Korean population, respectively (Figure 2). However, there was a trend towards significant difference in Caucasian population with a pooled OR of 1.08 (95% CI: 1.00-1.16, Z = 1.97, P = 0.05) (Figure 2). The sensitivity analysis showed that the results were not being driven by any one specific study, and the funnel plot did not detect there was evidence of publication bias (Figure 3).

**Table 2:** **Pooled genotype distribution of Val66Met polymorphism in Caucasian and Oriental population**

**Population**	**BPD patients**			**Health controls**		
	**Val/Val**	**Val/Met**	**Met/Met**	**Val/Val**	**Val/Met**	**Met/Met**
Caucasian	0.654	0.320	0.026	0.642	0.324	0.034
Oriental	0.296	0.486	0.218	0.311	0.489	0.200

**Figure 4:**
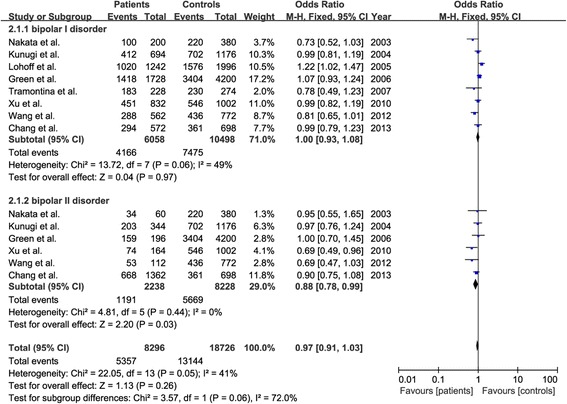
**Forest plot of association between BDNF gene Val66Met polymorphism and subtyped bipolar disorders.**

**Figure 5:**
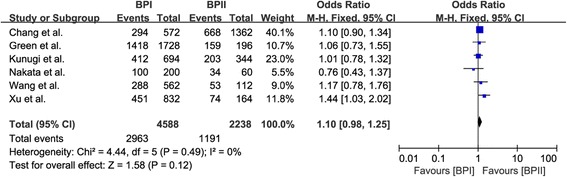
**Forest plot of comparison between bipolar I disorder and bipolar II disorder.**

Among 21 included studies, six case–control association studies made a distinction for clinical phenotypes between bipolar I disorder (BP I) and bipolar II disorder (BP II), and two other studies only recruited BP I patients (Table 3). A further meta-analysis of the data from the aforementioned eight studies did not find a significant difference in allelic distribution of Val66Met polymorphism between BP I patients and healthy controls with a pooled OR of 1.00 (95% CI: 0.93-1.08, Z = 0.04, P = 0.97) (Figure 4). However, there was a significant difference in allelic distribution of Val66Met polymorphism between BP II patients and healthy controls with a pooled OR of 0.88 (95% CI: 0.78-0.99, Z = 2.20, P = 0.03) (Figure 4). A post-hoc analysis did not find a significant difference in allelic distribution of Val66Met polymorphism between BP I patients and BP II patients with a pooled OR of 1.10 (95% CI: 0.98-1.25, Z = 1.58, P = 0.12) (Figure 5).

**Table 3:** **Descriptive characteristics of included association studies between BDNF gene Val66Met polymorphism and subtyped bipolar disorders**

**Study**	**Year**	**Ancestry**	**Diagnostic criteria**	**Patient’s phenotype**	**Cases**	**Controls**	**Case Val**	**Controls Val**
**Bipolar I disorder**								
Nakata et al. [39]	2003	Japanese	DSM-IV	BPI	100	190	118	220
Kunugi et al. [40]	2004	Japanese	DSM-IV	BPI	347	588	412	702
Lohoff et al. [43]	2005	Caucasian	DSM-IV	BPI	621	998	1020	1576
Green et al. [46]	2006	Caucasian	DSM-IV	BPI	864	2100	1418	3404
Tramontina et al. [48]	2007	Caucasian	DSM-IV	BPI	114	137	183	230
Xu et al. [54]	2010	Han Chinese	DSM-IV	BPI	416	501	451	546
Wang et al. [56]	2012	Han Chinese	DSM-IV	BPI	281	386	288	436
Chang et al. [30]	2013	Han Chinese	DSM-IV	BPI	286	349	294	361
**Bipolar II disorder**								
Nakata et al. [39]	2003	Japanese	DSM-IV	BPII	30	190	34	220
Kunugi et al. [40]	2004	Japanese	DSM-IV	BPII	172	588	203	702
Green et al. [46]	2006	Caucasian	DSM-IV	BPII	98	2100	159	3404
Xu et al. [54]	2010	Han Chinese	DSM-IV	BPII	82	501	74	546
Wang et al. [56]	2012	Han Chinese	DSM-IV	BPII	56	386	53	436
Chang et al. [30]	2013	Han Chinese	DSM-IV	BPII	681	349	668	361

## Discussion

With a total of 7,219 patients and 9,832 control cases, our meta-analysis included an additional 4,076 BPD cases and 3,485 healthy controls compared to a previous meta-analysis of case–control studies [22]. Similar to this previous meta-analysis, we did not find significant associations between the Val66Met polymorphism and BPD susceptibility either in a combined population or a subgroup of Oriental patients. However, there was a trend towards significant association in Caucasian population (fixed-effects pooled OR = 1.08, P = 0.05). Subgroup meta-analyses also showed that the Val allele may be a protective factor for BP II (fixed-effects pooled OR = 0.88, P = 0.03).

Our overall finding, that no convincing evidence for association between the Val66Met polymorphism and BPD as a whole, is consistent with a previous meta-analysis reported by Kanazawa et al. [22], which included 11 case–control designed studies up to February 2006. However, our overall finding is inconsistent with the result of a meta-analysis conducted by Fan et al. [15], which included all original case–control and family-based association studies published up to May 2007 and found a modest but statistically significant association between the Val allele and BPD susceptibility [15]. Both our analysis and the analysis of Kanazawa et al. [22] exclusively included case–control studies. In contrast, the analysis of Fan et al. [15] included five family-based association studies with a total of 858 families and a genome-wide association study with 1866 BPD patients and 2932 controls. The inclusion of different studies in these meta-analyses may explain the discrepancy among these three meta-analysis studies. It is well known that BPD is a highly inheritable disorder. Inclusion of family-based association studies in the meta-analysis of Fan et al. [15] might increase the probability of detecting difference between patients and controls.

The finding of insignificant association between the Val66Met polymorphism and BPD in Oriental population populations (Chinese, Japanese and Korean populations), but a trend towards significance in Caucasian population suggest that ethnic heterogeneity may affect the results of these genetic association studies. Fan et al. [15] reported that the allele frequencies of the Val66Met polymorphism in BDNF gene across individual studies and four HapMap populations (European, Chinese, Japanese and Yoruban populations) had significant global variations, which raised concerns of possible population stratification among case–control studies [15]. A more recent population genetic study found that there were substantial variations in BDNF coding regions and haplotype frequencies between 58 global populations with the Met allele of Val66Met ranged from 0-72% frequencies [58]. As previously pointed out, unless all study participants are from a homogenous ethnic group, the confounding effect from different ethnic groups is inevitable [10]. Moreover, control selection (e.g. healthy control or family control) might also result in the discrepancy of findings in these genetic association studies. A significant discrepancy between the pooled ORs from case–control studies and family-based studies raised a concern regarding a more generalized transmission distortion at this locus that is not disease related [15].

The finding of significant associations between Val66Met polymorphism and BP II suggests that the strength of association between Val66Met polymorphism and BPD could depend on the clinical phenotypes or subtypes of BPD such as with rapid cycling course, early onset, or substance comorbidity [10,28,30-32,34,50,55,59]. Interestingly, the Val allele may have opposite associations with disease susceptibility in different bipolar subtypes. The findings of two previous studies in Caucasian population (a case–control study and a family-based study) appeared that the Val allele was associated with an increased disease risk for rapid-cycling bipolar disorder (RCBD) [46,60], but this meta-analysis showed a decreased risk for BP II (especially in Han Chinese population). However, another family-based association analysis in Caucasian population did not replicate a significant association between the Val66Met polymorphism and BPD or RCBD. Thus the discrepancy of findings in these studies may stem from the differences of clinical phenotypes, ethnic origin and control selection.

Several limitations of this meta-analysis should be considered. One important limitation is that we only investigated relatively well-studied polymorphic variations in BDNF gene. Another limitation is that family-based studies and genome-wide association studies were excluded due to the heterogeneity of research methods. In a previous meta-analysis, the pooled OR derived from nine case–control studies is nominally significant (random-effects pooled OR = 1.07, P = 0.04), while the pooled OR derived from five family-based studies increases notably to an OR of 1.54 (P = 0.000019). Finally, since we primarily designed the current analysis to demonstrate the potential association(s) between Val66Met polymorphism and bipolar diagnostic boundaries, the association of this polymorphism with other characteristics of BPD such as sex, onset age, comorbidity, impairment in brain morphology and function, or treatment response, were not explored.

## Conclusions

Taken together, the Val66Met polymorphism in BDNF gene may be involved in the pathogenesis of BPD by influencing the susceptibility of specific subtypes such as BP II, but there is no compelling evidence of BDNF gene playing an important role in susceptibility to BPD across different ethnicities. The associations observed in current meta-analysis should be interpreted with caution. Further large-scale studies with same definitions of phenotypes and controls in homogenous ethnic groups are warranted to elucidate the relevance of BDNF gene variations as a risk factor for BPD (or diagnostic subtypes) susceptibility.
